# Mechanistic divergence between SOS response activation and antibiotic-induced plasmid conjugation in *Escherichia coli*

**DOI:** 10.1128/spectrum.00090-25

**Published:** 2025-05-28

**Authors:** Ruoxuan Zhao, Arkadiusz Nawrocki, Jakob Møller-Jensen, Gang Liu, John Elmerdahl Olsen, Line Elnif Thomsen

**Affiliations:** 1 Department of Veterinary and Animal Sciences, Faculty of Health and Medical Sciences, University of Copenhagen 53139 , Copenhagen, Capital Region of Denmark, Denmark; 2 Department of Biochemistry and Molecular Biology, University of Southern Denmark 123256 , Odense, Region Syddanmark, Denmark; 3 College of Veterinary Medicine, Qingdao Agricultural University 98431, Qingdao, Shandong, China; University of Manitoba, Winnipeg, Manitoba, Canada

**Keywords:** SOS response, conjugation, antibiotics, *Escherichia coli*, IncI1

## Abstract

The SOS response is a critical DNA damage repair mechanism in bacteria, designed to counteract genotoxic stress and ensure survival. This system can be activated by different classes of antimicrobial agents, each inducing the SOS response through different mechanisms. Moreover, it has been observed that certain antibiotics can enhance conjugative plasmid transfer frequencies. However, while previous studies have suggested that the SOS response contributes to horizontal transfer of certain genes, its role in plasmid conjugation remains unclear. In this study, we investigated the relationship between the SOS response and conjugation of IncI1 and IncFII plasmids harboring various *bla_CTX-M_* resistance genes. Results showed that cefotaxime and mitomycin C induced both the SOS response and conjugation, while ciprofloxacin induced the SOS response without affecting conjugation frequencies. Further analysis of SOS mutants, ranging from constitutively inactive to hyper-induced states, revealed no correlation between SOS levels and conjugation frequencies, despite upregulation of *tra* gene expression in a SOS hyper-induced strain. Proteomic analysis revealed that cefotaxime-induced conjugation was associated with increased transfer and pilus protein expression. In contrast, the SOS hyper-induced strain displayed limited upregulation of plasmid-encoded proteins, suggesting post-transcriptional regulation. Additionally, putative LexA binding sites on the IncI1 plasmid revealed potential SOS-mediated regulation of plasmid genes, highlighting the interaction between the SOS response and plasmid, although it did not significantly affect conjugation.

**IMPORTANCE:**

Plasmids play a critical role in the dissemination of antibiotic resistance through conjugation. Recent research suggests that the use of antibiotics not only selects for already resistant variants but further increases the rate of plasmid-encoded conjugative transmission by increasing expression of the conjugative system. At the same time, these antibiotics are known to induce the stress-related SOS response in bacteria. To be able to counteract an antibiotic-induced increase in conjugative transfer of resistance plasmid, there is a need for a fundamental understanding of the regulation of transmission, including whether this happens through activation of the SOS response. In this research, we show that antibiotic-induced conjugation and induction of the SOS response happen through different mechanisms, and thus that future strategies to control the spread of antibiotics cannot interfere with the SOS response as its target.

## INTRODUCTION

The SOS response is a conserved DNA damage repair mechanism among prokaryotes that mitigates genotoxic damage and ensures survival. The regulon encompasses over 48 genes in *Escherichia coli* that are pivotal for DNA repair processes, potentially resulting in recombination and mutagenesis (1, 2). During unstressed conditions, LexA serves as a transcriptional repressor for the SOS regulon by binding to specific sequences, known as LexA-boxes or SOS-boxes. However, upon exposure to genotoxic stresses such as UV irradiation or antibiotic treatment, the accumulation of single-stranded DNA (ssDNA) occurs. This accumulation, in association with RecA and ATP, led to the formation of RecA–ssDNA–ATP filaments (RecA*), triggering autocleavage of LexA, thereby de-repressing the SOS regulon genes and initiating the DNA repair process (3). The *sulA* gene, regulated by LexA, acts as a cell division inhibitor that provides cells with the necessary time to repair damage. SOS effectors, such as *uvrA*, which is responsible for nucleotide excision repair, and *recA*, which plays a pivotal role in homologous recombination, facilitate high-fidelity repair of DNA damage. Nonetheless, in scenarios of sustained and severe damage, RecA* activates the expression of error-prone polymerase genes *umuDC* or *dinB* to facilitate DNA replication across genomic lesions, consequently elevating the rate of mutagenesis (4, 5).

The conjugative transfer of plasmids between bacteria represents a form of horizontal gene transfer and serves as a critical mechanism for the dissemination of antibiotic resistance genes. This process relies on the expression of transfer (*tra*) and pilus (*pil*) genes, which facilitates the formation of the type IV secretion system necessary for plasmid transfer (6). Our recent review highlighted prior results showing that various antimicrobial agents can induce conjugation in different bacterial species (6). However, the ability of antibiotics to induce conjugation appears to differ depending on the class of the antimicrobial agent and the resistance gene carried by the plasmid (7). Furthermore, several classes of antimicrobials are able to induce the bacterial SOS response either directly by targeting DNA replication or repair (e.g., fluoroquinolones and mitomycin C) or indirectly by targeting other pathways that cause DNA damage (e.g., β-lactams and aminoglycosides) (8–10). This raised the question of whether the SOS response and accelerated plasmid conjugation are connected through the SOS-mediated upregulation of plasmid transfer (*tra*) genes, or if they are independent responses to antibiotic stress. In this study, we aimed to investigate the relationship between the SOS response and conjugation induced by the antibiotics cefotaxime, ciprofloxacin, and mitomycin C. By examining the effects of SOS-inducing compounds on plasmid conjugation frequencies in mutant strains with defective or hyperactive SOS responses, we conclude that the SOS response and the induced increase in conjugation are unrelated in *E. coli* MG1655.

## RESULTS

### Antimicrobial agents induced the SOS response and conjugation

**Fig 1:**
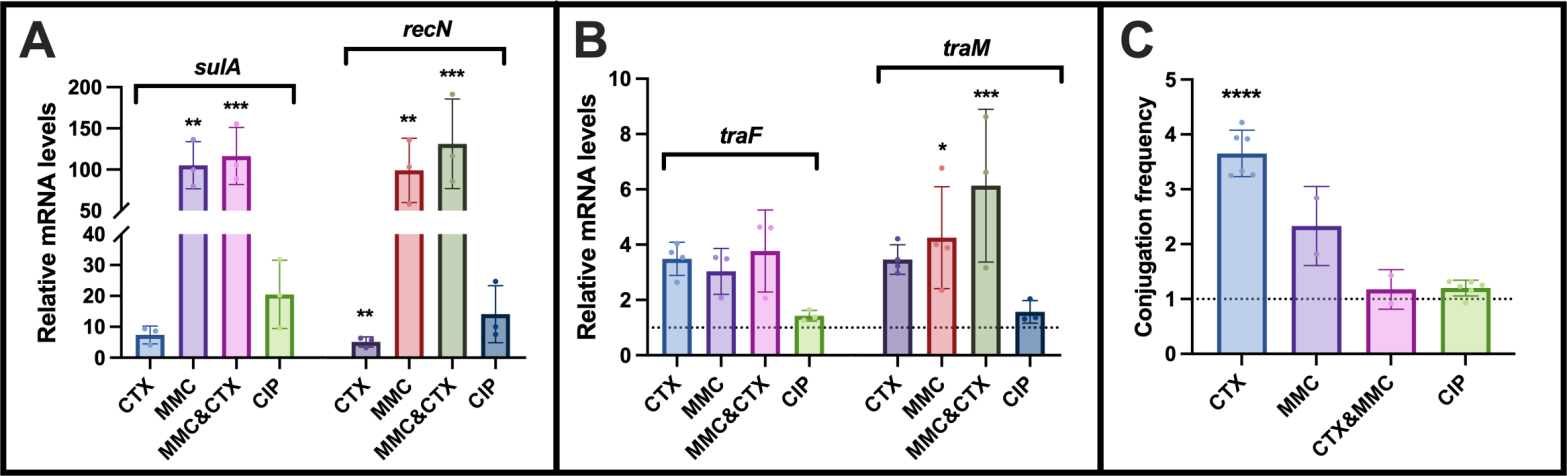
Investigation of SOS response, *tra*-gene expression, and conjugation when WT is exposed to sub-inhibitory concentrations of antimicrobial agents (½-MIC of CTX [128 µg/mL], ½-MIC of MMC [2 µg/mL], combination of 1/8-MIC of CTX [32 µg/mL] and ¼-MIC of MMC [1 µg/mL], and ½-MIC of CIP [0.004 µg/mL]). (**A**) Antimicrobials induce bacterial SOS response to varying degrees. (**B**) CTX and MMC, as well as their combined treatment, lead to the upregulation of *tra*-genes expression, whereas CIP treatment has no effect on *tra* expression. (**C**) CTX and MMC up-regulated the conjugation frequency of pTF2, whereas CTX+MMC and CIP treatment did not affect conjugation. Data are presented as fold change relative to WT without antibiotic treatment (dotted line), with the values presented as average plus standard deviation. Each dot in the visual representation corresponds to an individual biological replicate. The fold changes were compared to WT without antibiotic treatment to analyze the differences: *P ≤ 0.05, **P ≤ 0.01, ***P ≤ 0.001, and ****P ≤ 0.0001.

Previously, we have demonstrated that cefotaxime (CTX), a β-lactam antibiotic, significantly enhanced the transfer frequency of pTF2, an IncI1 plasmid harboring the *bla_CTX-M-1_* resistance gene (11). In addition, it has been suggested that different classes of antimicrobials are able to induce the SOS response (8–10). To investigate the association between an induced SOS response and plasmid conjugation, we exposed *E. coli* MG1655/pTF2 (WT) to CTX, and to ciprofloxacin (CIP) and mitomycin C (MMC), as both are known to cause DNA lesions and thereby induce the SOS response in *E. coli* (12). Minimal inhibitory concentration (MIC) was determined for all three antibiotics: CTX (256 µg/mL), CIP (0.008 µg/mL), and MMC (4 µg/mL). WT was grown without and with ½-MIC of each antibiotic, as well as a combination of 1/8-MIC of CTX and ¼-MIC of MMC. This revealed that within the first 4 h, the growth was only slightly affected, but MMC and MMC+CTX exposure led to an early entry into the stationary phase compared to untreated WT (Fig. S1). Samples treated with sub-inhibitory concentrations of antibiotics were collected at mid-exponential phase for quantitative PCR (qPCR) analysis and conjugation experiments. CTX exposure induced the SOS response, as evidenced by an average of a fivefold increase in the expression of the two SOS-regulated genes, *sulA* and *recN* (Fig. 1A). CIP, a fluoroquinolone that targets DNA gyrase, led to a 14- and 20-fold increase in the expression of *sulA* and *recN*, respectively. In contrast, MMC, a potent DNA crosslinker, hyper-induced the SOS response, resulting in approximately a 100-fold increase in the expression of both analyzed SOS genes (Fig. 1A). CTX and MMC treatment also induced the transcription of the transfer genes *traF* and *traM* (Fig. 1B), and this induction resulted in elevated conjugation frequencies (Fig. 1C). Interestingly, the hyperinduction of the SOS response elicited by MMC led to increased conjugation frequencies comparable to the level induced by CTX, despite CTX eliciting only a minor SOS response (Fig. 1C). With a combined treatment using both CTX and MMC, we observed high induction of the SOS response and upregulated *tra* gene expression (Fig. 1A and B), but the conjugation frequency was not significantly affected by the treatment when compared to untreated bacteria (Fig. 1C). CIP exposure induced the SOS response to a degree intermediate between that of CTX and MMC (Fig. 1A). However, this did not lead to the induction of the *tra* gene expression and conjugation (Fig. 1B and C).

CTX was observed to increase conjugation frequencies with minimal induction of the SOS response, while CIP triggered the SOS response but did not alter conjugation frequencies. To determine whether the observed differences between the effects of CTX and CIP were due to the WT strain’s resistance to CTX and sensitivity to CIP, we investigated the SOS response and *tra* gene expression in six *E. coli* MG1655/pTF2 strains (RX63-RX71) with varying levels of CIP resistance. The results showed that the SOS response was downregulated in the CIP-resistant strains following CIP treatment, but uncorrelated to resistance levels, whereas *tra* gene expression remained unaffected (Fig. S2). These findings suggested that the effect of CIP treatment on SOS response and *tra* gene expression is independent of the strain’s resistance level.

### SOS response levels are uncorrelated with plasmid conjugation frequencies

**Fig 2:**
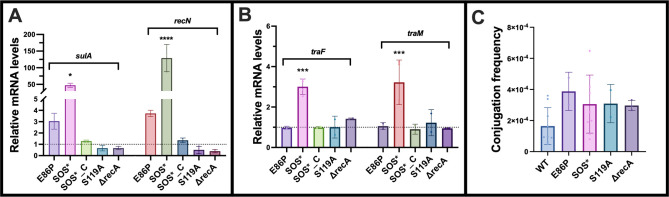
(**A**) SOS response gene expression levels and (**B**) *tra*-gene expression levels in SOS variants relative to WT. Data are presented as fold change relative to WT (dotted line), and the values are presented as average plus standard deviation. (**C**) Conjugation frequency of SOS mutants. Each dot in the visual representation corresponds to individual biological replicates. The stars indicate statistical significance between the mutants and the WT at different levels: *P ≤ 0.05, ***P ≤ 0.001, and ****P ≤ 0.0001.

In the initial experiments using CTX, MMC, and CIP, we found no direct correlation between the level of the SOS response and conjugation. To explore this further, we constructed a series of SOS mutants through genetic engineering of the two SOS response regulators, RecA and LexA, creating strains with SOS responses ranging from constitutively inactive to constitutively hyper-induced. This series included two strains with inactivated SOS response: the Δ*recA* strain, which eliminates the LexA auto-cleavage inducer, and the LexA S119A mutant (S119A), which is deficient in auto-cleavage. As expected, SOS gene expression was reduced compared to WT (Fig. 2A). Included were also two mutants with induced SOS response: LexA E86P (E86P), carrying a mutation which increases the rate of LexA auto-cleavage, and SOS*, which spontaneously occurred during the construction of the mutants. The expression of *sulA* and *recN* was threefold to fourfold upregulated in E86P, compared to the WT (Fig. 2A). Using whole-genome sequencing, the SOS* strain was shown to contain four single nucleotide polymorphisms (SNPs) located in *lexA* (E74V, S119A, P176L, and I188T), one SNP in *nuoC* (D102G), *gsiD* (Y70D), and a deletion of nucleotide 1486 in *yflW*. This resulted in hyper-induction of SOS gene expression, with *sulA* and *recN* being upregulated 47- and 128-fold, respectively, compared to WT (Fig. 2A). This elevated SOS response did not impact bacterial growth and only caused a slight increase in bacterial size (Fig. S1). Complementation of the SOS* strain with the WT *lexA* gene (SOS*_C) restored the bacterial SOS response to WT levels (Fig. 2A), supporting that the *nuoC, gsiD,* and *yflW* mutations did not affect SOS expression levels.

To explore the impact of the different levels of the SOS response on plasmid conjugation frequency, we conducted a comparative analysis of transcription levels of *tra*-genes and conjugation frequencies of all SOS mutant strains. Both SOS-inactivated strains (Δ*recA* and S119A) and the mildly SOS-induced strain E86P exhibited *tra* gene expression levels similar to WT. In contrast, the hyper-induced SOS response strain SOS* displayed an approximately threefold increase in *tra* gene expression relative to WT, a magnitude of upregulation comparable to the upregulation induced by sub-inhibitory concentrations of CTX and MMC in WT (Fig. 1B). Plasmid copy number analysis rejected the possibility that the upregulation of *tra* gene expression in SOS* was due to an increase in plasmid copy number (Fig. S3).

Upon examining the conjugation frequencies of the four SOS mutants, no significant difference was observed compared to WT, despite the observed increase in *tra* gene expression in SOS* (Fig. 2C).

To analyze whether the findings applied to other plasmid Inc-groups or were affected by differences in CTX-M genes, we tested two additional plasmids in the WT and SOS-mutant backgrounds: an IncFII plasmid carrying *bla_CTX-M-14_* (RX121-125) and an IncI1 plasmid carrying *bla_CTX-M-55_* (RX126-130). Consistent with the results of pTF2, CTX was able to induce conjugation frequencies of the two plasmids, whereas the conjugation frequencies were not affected by the SOS levels (Fig. S4).

### Proteomics analysis

**Fig 3:**
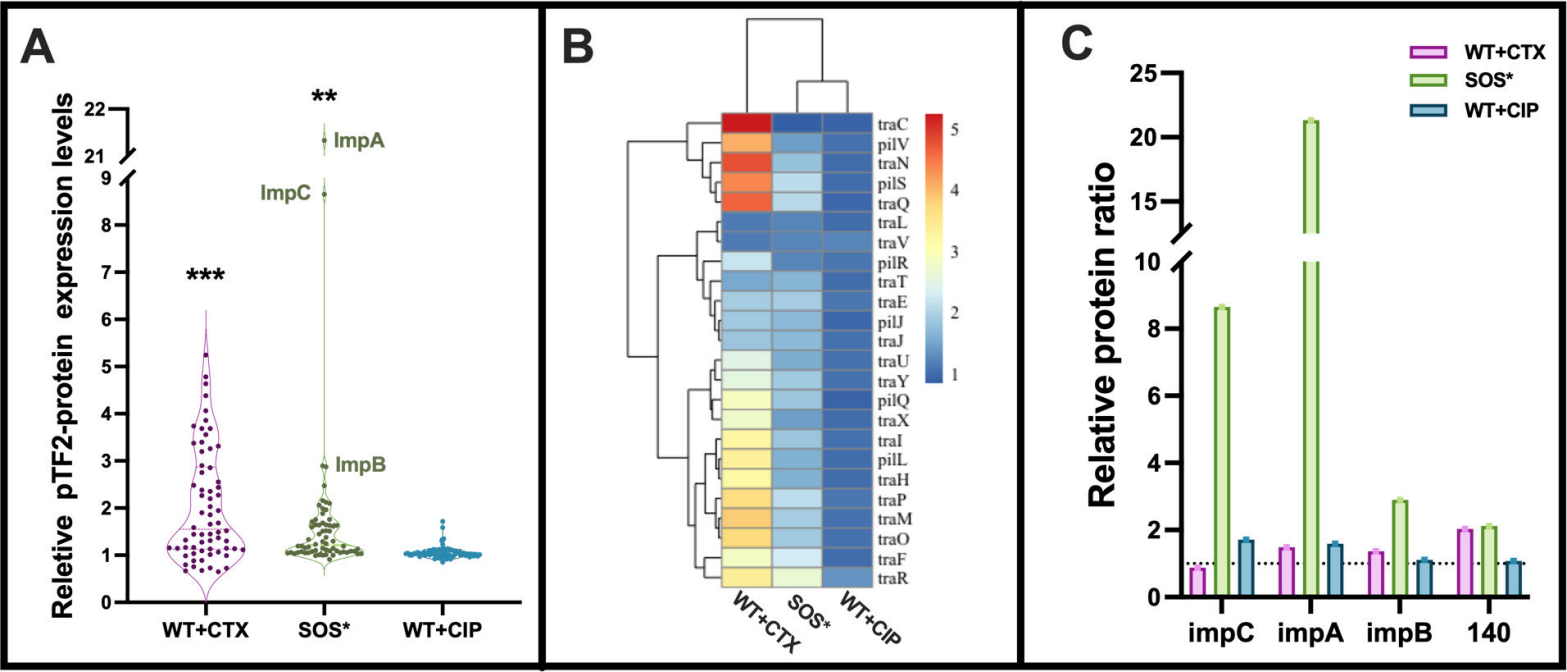
Proteomic analysis of SOS* and the WT ± ½-MIC of CTX or CIP. The data are presented as fold change relative to WT without antibiotic treatment. (**A**) The violin plot demonstrates a relative abundance of identified proteins encoded by plasmid pTF2. Each dot represents the abundance ratio of a specific identified protein. The relative protein expression levels were compared to WT without antibiotic treatment to analyze the differences: **P ≤ 0.01 and ***P ≤ 0.001. (**B**) The heatmap of the relative abundance of conjugation-related proteins. (**C**) Relative protein ratio of the four pTF2-encoded proteins up-regulated in SOS*.

The elevated *tra* gene expression in SOS* was not reflected in increased conjugation levels, which prompted us to investigate the protein expression in the two strains. The proteomic analysis was performed on WT, SOS*, and WT exposed to ½ -MIC of either CTX or CIP. This analysis identified a total of 2,974 individual proteins, of which 66 proteins were encoded by the plasmid pTF2 (Table S1), while the remaining 2,908 were encoded by the chromosome (repository DOI:10.17894/ucph.b4a308f9-6138-486e-9b46-f935011c3300). CTX treatment of WT increased the expression levels of 27 plasmid-encoded proteins, including 18 conjugation-related proteins, by twofold to fivefold (Fig. 3A and B). In contrast, the hyper-induced SOS response caused a limited increase in the expression of pTF2-proteins compared to untreated WT. Of these, nine proteins were up-regulated more than twofold, while most others showed an approximate 1.5-fold increase (Fig. 3A). Notably, two proteins, ImpA and ImpC, part of the pTF2-encoded *impCAB* operon, exhibited significant up-regulation in SOS*, with relative protein ratios peaking at 9 and 21, respectively. Additionally, ImpB and a hypothetical protein HTE32_RS00140 (hereafter called 140) were up-regulated twofold in SOS*, indicating regulation by LexA (Fig. 3C). CTX treatment of the WT elevated Tra protein levels compared to the SOS* strain. Conversely, CIP did not induce significant protein expression from pTF2 (Fig. 3A and B), consistent with the lack of CIP-induced *tra* gene expression and conjugation (Fig. 1B and C). Enrichment analysis of chromosomally encoded proteins in SOS* revealed an upregulation of the SOS regulon (repository DOI:10.17894/ucph.b4a308f9-6138-486e-9b46-f935011c3300). In contrast, WT+CTX versus WT did not reveal a significant change in the SOS response, and the limited number of differentially expressed proteins in WT+CIP versus WT was too few to enable enrichment analysis.

### SOS box prediction on plasmid pTF2

The *lexA* mutation in the SOS* strain led to the upregulation of SOS regulon proteins, and the upregulated pTF2-encoded proteins were also potentially regulated by LexA. Previously, LexA was shown to regulate at least 48 chromosomal genes in *E. coli*, with the LexA-box identified as 5′-TA**CTG**(TA)_5_**CAG**TA-3′ (2). Notably, the base pair spacer sequence within the conserved regions (bold sequence) influences the binding affinity of LexA. To identify potential LexA-binding sites on pTF2, three search patterns were employed: **CTG**NNTNNNNNNN**CAG**, **TTG**DNTDNNHNNH**CAG**, and **CTG**DNTDNNHNNH**CAA**, with regions located near a putative initiation codon (−200 to +40 nucleotides) as the filter. This analysis identified 10 potential SOS-boxes that regulate nine genes on pFT2 (Table 1). Among these, two LexA binding sites situated in the promoter region of the *impCAB* operon and one site in the promoter region of *dqlB* have relatively higher LexA binding affinity, with heterology index (HI) values less than 15 (12). The observed upregulation of ImpCAB protein expression supported a direct regulation by LexA (Fig. 3C). DqlB was, however, not identified in the proteomic analysis. The *dqlB* is a DinQ-like type I toxin, and previous results have shown that *dinQ* expression is regulated by LexA. However, it is also regulated at post-transcriptional levels, which might explain the lack of changes in DqlB protein levels (13). Additionally, three *tra*-genes that were identified as having a putative SOS-box (*traN*, *traQ,* and *traU*) also showed a minor increased expression at the protein level. However, since their HI values exceeded 15, it is unlikely that LexA directly regulates those transfer genes.

**TABLE 1:** Potential LexA-binding sites on pTF2

Locus tag	Gene name	Distance^*a*^	Sequence^*b*^	HI^*c*^
HTE32_RS00475	*impCAB*	−21	TA**CTG**TATATACATA**CAG**CA	1.60
HTE32_RS00535	*dqlB*	−119	AC**CTG**TTTGTTCAGC**CAG**GA	11.64
HTE32_RS00475	*impCAB*	−48	AA**CTG**AATCCACACA**CAG**TC	13.49
HTE32_RS00185	*traS*	−8	CC**TTG**TATTGCCGAA**CAG**GA	17.90
HTE32_RS00370	*–^d^*	−196	TC**CTG**TGTTGCACCC**CAG**AC	20.28
HTE32_RS00070	*–*	+22	AC**CTG**GCTGACCCGG**CAG**TC	21.96
HTE32_RS00125	*trbA*	−14	GT**TTG**ATTTAAATTC**CAG**AA	22.79
HTE32_RS00210	*traN*	−130	GG**TTG**TCTGGCCGAC**CAG**AC	24.54
HTE32_RS00175	*traU*	−119	CC**CTG**ATTCTGACAC**CAG**AT	24.73
HTE32_RS00195	*traQ*	−105	GC**CTG**GATTCGCTGG**CAG**GG	26.13

^*a*^ The “distance” refers to the location of the predicted SOS-box relative to the ATG codon of the respective gene.

^*b*^ The underlined sequences represent deviations from the consensus core LexA binding site, and the gray shading base indicates that the site is mismatched with the LexA-box (5′-TA**CTG**(TA)_5_**CAG**TA-3′).

^*c*^ The heterology index values were calculated according to Levis’s model (12).

^*d*^ "–" indicates unannotated gene.

### SOS-induced plasmid-encoded proteins do not affect conjugation

**Fig 4:**
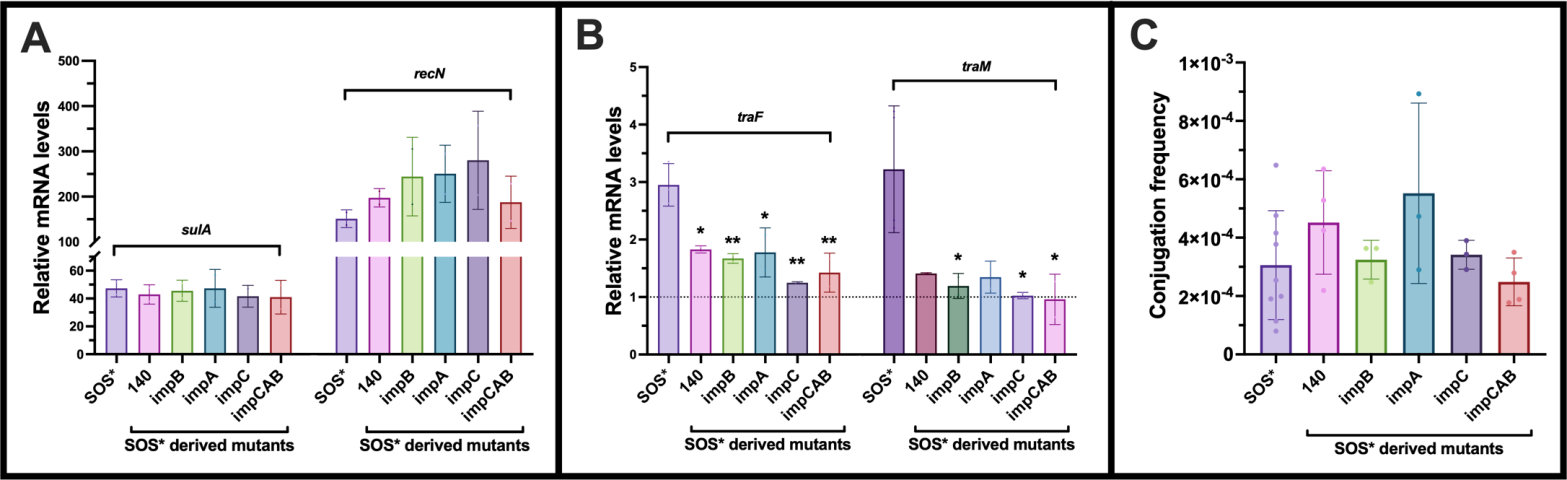
(**A**) Deletion of pTF2-encoded upregulated genes in SOS* did not affect SOS-response levels (*sulA* and *recN*) when compared to SOS*. (**B**) *tra* gene expression (*traF* and *traM*) was reduced in SOS*-derived mutants. (**C**) Conjugation frequency of SOS* and SOS*-derived mutants revealed no significant changes. The values are presented as average plus standard deviation, and the fold changes were compared to that of SOS* to analyze the differences: *P ≤ 0.05, **P ≤ 0.01.

The proteins ImpCAB and 140 were found to be upregulated in SOS* compared to WT. Previous studies have demonstrated that ImpCAB contributes to bacterial survival following UV radiation (14), whereas 140 is a hypothetical protein. Signal peptide predictions suggested that 140 is likely localized in the cytoplasm, and structure predictions indicated a mixed α/β domain in the C-terminal region, which may be associated with DNA/RNA-binding activity (Fig. S5). To investigate the impact of ImpCAB and 140 on conjugation frequency, we created single knock-out mutations of these genes in SOS*. Significant reduction in *tra* gene expression was seen, but this reduction was not reflected in significant changes in conjugation frequency (Fig. 4A through C).

## DISCUSSIONS

Despite extensive research on the SOS response mechanism, its connection to antimicrobial resistance remains incompletely understood. Recent findings have demonstrated that bacterial antibiotic resistance can be acquired or strengthened through the induction of the SOS response via different ways, including induction of mutagenesis, regulation of resistance genes, formation of persister cells, and biofilm development (10, 15, 16). The impact of the SOS response on the spread of resistance through conjugation has not been elucidated, but a study has suggested that fluoroquinolones, which are able to induce the SOS response, induce conjugation in *E. coli* (17). Meanwhile, it has been shown that the SOS response plays a role in antibiotic-induced horizontal gene transfer via transduction and dissemination of integrating conjugative elements (16, 18, 19).

In this study, we aimed to investigate the impact of the SOS response on conjugative transfer. When exposing *E. coli* MG1655/pTF2 to different antibiotics, we found that CTX, CIP, and MMC induced the SOS response 5-, 14-, and more than 100-fold, respectively. However, the levels of conjugation did not correlate directly with the SOS induction, as CTX treatment revealed higher levels of conjugation than MMC treatment, and CIP did not affect conjugation frequency. These results suggest that SOS induction does not affect conjugation.

As antibiotics have been shown to increase conjugation, we wanted to eliminate antibiotics in the investigation of the effect of the SOS response on conjugation. We therefore created four mutants which either prevented the initiation of the SOS response (Δ*recA* and S119A) or constitutively expressed the SOS response (E86P and SOS*). Analyzing these four mutants showed a similar uncorrelated pattern between SOS induction and conjugation. The medium (E86P) and highly expressed SOS (SOS*) mutants had similar levels of conjugation frequency as the two mutants with inactivated SOS response (Δ*recA* and S119A) and the WT. Further analysis of the four SOS mutants harboring the plasmids pCTX21 (IncI1) and pCTX22 (IncFII) also revealed no significant difference in conjugation frequencies compared to their corresponding WT. These data suggest that antibiotic-induced conjugation and induction of the SOS response are two independent responses to antibiotic treatment in *E. coli*.

To confirm that the level of SOS induction was unrelated to the resistance pattern of the WT, six CIP-resistant *E. coli* MG1655/pTF2 strains, with different MIC levels, were included. The results showed that the level of SOS induction by CIP treatment was decreased; however, it was uncorrelated with the level of resistance. Furthermore, the results revealed a similar lack of correlation between the level of SOS induction and the level of *tra* gene expression.

A proteomic analysis showed that CTX treatment of WT induced the levels of Tra proteins, supporting the increase in *tra* gene expression and conjugation. These data align with previous proteomic results, showing that CTX induced Tra proteins (11). CIP treatment did not induce Tra protein levels, supporting that *tra* gene expression and conjugation were not induced by CIP. Under two conditions with high levels of SOS induction, MMC+CTX treatment of WT or SOS*, it was observed that both the SOS response and *tra* gene expression were induced. However, under both conditions, the increase in *tra* gene expression did not lead to induced conjugation rates. In addition, despite SOS* showing significantly induced *tra* gene expression, the increase in Tra protein levels was limited. This suggests that *tra* gene expression may also be regulated on a post-transcriptional level in strains with induced SOS expression. The proteomic analysis showed that primarily the SOS response was upregulated in the SOS* strain, and it has been seen that mRNA stability can be reduced as a response to stress, to reduce the cost of protein synthesis (20). Furthermore, it has been shown in IncF plasmids that the regulatory *traJ* mRNA is a substrate for repression by small RNAs, thereby limiting translation and conjugation (21). Post-transcriptional regulation of the *tra* gene expression has also been shown in *Streptomyces lividans,* supporting this additional level of regulation of transfer genes (22).

The most highly induced pTF2-encoded proteins in SOS* were expressed from the *imp* operon. The *impAB* genes share homology with the *umuDC* operon, and ImpC shows similarity to DinI, genes which are part of the chromosomal SOS regulon (23, 24). The upregulation of the *imp* operon corresponds with the predicted SOS box in the promoter region of *impCAB*. The hypothetical gene 140 was up-regulated approximately twofold in the proteomic analysis but was not predicted to contain an SOS box. These up-regulated genes could potentially play a role in the SOS* phenotype; however, deleting the genes did not significantly affect SOS expression levels or conjugation frequencies in the SOS* background. Protein 140 was upregulated in WT exposed to CTX to a similar level, but not regulated when WT was exposed to CIP. As the SOS response was upregulated fivefold by CTX and 14-fold by CIP, it supports that gene 140 is not involved in the SOS response.

We have shown that different levels of the SOS response do not affect conjugation of two different IncI1 plasmids and one IncFII plasmid between two *E. coli* strains. Contrary to our results, Beaber et al. have shown that the induction of the SOS response by CIP or MMC induced the conjugation from *E. coli* to *Vibrio cholerae* (16). However, differences between bacterial strains, conjugative plasmids, and variations in how compounds affect the SOS response may exist. It has been shown that different drugs induce various aspects of the SOS response (25), which may affect horizontal gene transfer differently. Additional analysis is therefore needed to evaluate whether this is specific to *E. coli* or a more general phenomenon in other bacterial species.

The SOS response is induced in the recipient cell during conjugation, as a response to the transfer of ssDNA (26). Furthermore, it has been shown that when comparing conjugation rate, CIP treatment, and thereby SOS induction, donor leads to lower frequencies of conjugation, compared to only treatment of the recipient, although the highest levels of conjugation were observed when both donor and recipient were treated with CIP (27). This suggests that the effect of the presence of antibiotics during conjugation may increase conjugation due to the induction of the SOS response in the recipient. In our study, we eliminated the antibiotic effect on the recipient by pre-treating the donor and removing the antibiotics before conjugation. Furthermore, using SOS mutant strains, we eliminated the effect of the antibiotics on the process, making it possible to evaluate the effect of the SOS response of the donor on conjugation.

In conclusion, we found that in *E. coli* MG1655, containing conjugative plasmids, there was no correlation between the level of SOS induction of the donor strain and conjugation frequencies. Although the SOS response is widely acknowledged as a target for reducing spontaneous bacterial resistance and enhancing bacterial susceptibility to antibiotics (28, 29), evidence supporting its use as a target for limiting horizontal gene transfer remains insufficient.

## MATERIALS AND METHODS

### Bacterial strains and media

Bacterial strains and plasmids are shown in Table 2. Strains were grown in Luria-Bertani (LB) at 37°C, except strains containing plasmid pKD46, which were grown at 28°C. Media were supplemented with antibiotics when appropriate (Sigma, Denmark).

**TABLE 2:** Bacterial strains and plasmids used in this study

Number	Strain	Genotype or description	References
RX47	WT	*E. coli* MG1655 + *bla*_CTX–*M–*1_ containing IncI1 plasmid pTF2 (CTX^R^)	(11)
RX63		LM693/pTF2 (CIP^R^, CTX^R^): *gyrA1* S83L, *gyrA2* D87N, *parC* S80I	(30), this study
RX67		LM707/pTF2 (CIP^R^, CTX^R^): *gyrA1* S83L, *gyrA2* D87N, *parC* S80I, ∆*marR*	(30), this study
RX68		LM703/pTF2 (CIP^R^, CTX^R^): *gyrA1* S83L, *parC* S80I, ∆*marR*, ∆*acrR*	(30), this study
RX69		LM875/pTF2 (CIP^R^, CTX^R^): *gyrA1* S83L, *gyrA2* D87N, *parC* S80I, ∆*acrR*	(30), this study
RX70		LM878/pTF2 (CIP^R^, CTX^R^): *gyrA2* D87N, *parC* S80I, ∆*marR*, ∆*acrR*	(30), this study
RX71		LM705/pTF2 (CIP^R^, CTX^R^): *gyrA1* S83L, *gyrA2* D87N, *parC* S80I, ∆*marR*, ∆*acrR*	(30), this study
LG97	FRT_Kan	Kan resistance cassette inserted downstream of the *lexA* gene, intermediate in the construction of *lexA* mutants	This study
RX148	FRT_Cm	Cm resistance cassette inserted downstream of the *lexA* gene, intermediate in the construction of SOS*_C	This study
RX110	E86P	MG1655 *lexA* (E86P)/pTF2	This study
RX106	SOS*	MG1655 *lexA* (E74V, S119A, P176L, I188T)/pTF2	This study
RX153	SOS*_C	SOS* + WT *lexA*/ pTF2	This study
RX104	S119A	MG1655 *lexA* (S119A)/pTF2	This study
RX108	∆*recA*	MG1655 ∆*recA*/pTF2	This study
RX155	140	SOS* ΔHTE32_RS00140/pTF2	This study
RX145	impB	SOS* Δ*impB*/pTF2	This study
RX151	impA	SOS* Δ*impA*/pTF2	This study
RX152	impC	SOS* Δ*impC*/pTF2	This study
RX146	impCAB	SOS* Δ*impCAB*/pTF2	This study
	J53-2	*E. coli*, Rif^R^	(31)
	ATCC 25922	Reference strain for MIC measurement according to the CLSI guideline	(32)
RX121	WT/pCTX22	*E. coli* MG1655 + *bla*_CTX–*M–14*_ containing IncFII plasmid pCTX22 (CTX^R^)	(33), this study
RX122	E86P/pCTX22	MG1655 *lexA* (E86P)/pCTX22	(33), this study
RX123	Δ*recA*/pCTX22	MG1655 Δ*recA*/pCTX22	(33), this study
RX124	S119A/pCTX22	MG1655 *lexA* (S119A)/pCTX22	(33), this study
RX125	SOS*/pCTX22	MG1655 *lexA* (E74V, S119A, P176L, I188T)/pCTX22	(33), this study
RX126	WT/pCTX21	*E. coli* MG1655 + *bla*_CTX–*M–55*_ containing IncI1 plasmid pCTX21 (CTX^R^)	(33), this study
RX127	Δ*recA*/pCTX21	MG1655 Δ*recA*/pCTX21	(33), this study
RX128	E86P/pCTX21	MG1655 *lexA* (E86P)/pCTX21	(33), this study
RX129	SOS*/pCTX21	MG1655 *lexA* (E74V, S119A, P176L, I188T)/pCTX21	(33), this study
RX130	S119A/pCTX21	MG1655 *lexA* (S119A)/pCTX21	(33), this study
Plasmids			
	pKD46	rep_pSC101_^ts^ Gen^R^ P_araBAD_γβ *exo*	(34)
	pKD3	rep_R6K_ γAmp^R^ FRT Cm^R^ FRT	(35)
	pKD4	rep_R6K_ γAmp^R^ FRT Kan^R^ FRT	(35)

### Construction of mutants

Primers used for mutant constructions are listed in Table S2. Construction of the Δ*recA* mutant was performed by replacing the gene with a kanamycin (Kan) resistance cassette, using the Lambda-red recombinase system (35). For the construction of *lexA* mutants, the Kan resistance cassette was inserted into the non-coding region between *lexA* and *dinF* in the WT strain, creating strain FRT_Kan. This intermediate mutant strain served as a template for introducing point mutations in *lexA*. For *lexA* complementation of the SOS* strain, a chloramphenicol (Cm) resistance cassette was amplified from pKD3 and inserted downstream of *lexA* in WT, generating another intermediate mutant, strain FRT_Cm. The WT *lexA* sequence, together with its downstream Cm resistance gene, was amplified from strain FRT_Cm and inserted into the chromosome of SOS*.

The knockout of genes *impA*, *impB*, *impC*, *impCAB,* and *140* in SOS* was performed by replacing the genes with a Cm resistance cassette using the lambda-red recombinase system.

All constructed mutants were verified through PCR and Sanger sequencing (Macrogen, Netherlands). Whole-genome sequencing of SOS* was performed using the MiSeq platform (Illumina, University of Copenhagen) with a Nextera XT v3 kit (Illumina), as previously described (36).

### Minimal inhibitory concentration determination

The MIC of each strain against CTX, CIP, and MMC was determined using broth microdilution methods (32). CTX and MMC interactions were assessed using the checkerboard assay as previously described (37). *E. coli* ATCC 25922 served as a control, and MIC was measured in two biological replications with three technical replications.

### Growth analysis

Overnight cultures were diluted to an optical density at 600 nm (OD_600_) of 0.05 in 10 mL LB with or without sub-inhibitory concentrations of antimicrobial agents and grown at 37°C (180 rpm), and OD_600_ was measured over 24 h. The growth experiments were performed in two biological replications.

### Conjugation frequency investigation

WT, SOS mutant variants, and SOS* mutants were used as donors, and *E. coli* J53-2 was used as the recipient. Cultures without and with ½ MIC antibiotic treatment were collected at mid-exponential phase. Bacteria were washed (5,000 × *g*, 7 min, room temperature) to remove residual antimicrobials. OD_600_ value of each culture was adjusted to 1 in LB, and 500 µL of donor and 500 µL of recipient cells were mixed and statically incubated at 37°C for 1 h. Following incubation, the mixtures were serially diluted and plated on LB agar plates containing 2 µg/mL CTX (quantifying donors + transconjugants), and 2 µg/mL CTX and 50 µg/mL rifampicin (quantifying transconjugants only).

### Real-time quantitative PCR and relative plasmid copy number estimation

Strains were grown as described for conjugation. Approximately 8 × 10^8^ cells per group were harvested, and total RNA was extracted using the FastPrep cell disrupter system (Qbiogene, France) and RNeasy Mini Kit (Qiagen, Sweden). The RNA quantity was determined using a NanoDrop 1000 spectrophotometer (Thermo Scientific, Denmark). Subsequent steps involved the removal of DNA and reverse transcription of mRNA into cDNA using PrimeScript RT Reagent Kit with gDNA Eraser (TAKARA Bio, Kusatsu, Japan). Real-time quantitative PCR (RT-qPCR) analysis was performed on a LightCycler 96 system (Roche, Denmark) using FastStart Essential DNA Green Master (Roche, Denmark) and primers listed in Table S2. The transcript levels were normalized to the validated reference gene *gapA*, and the relative gene expression level was calculated according to the 2^−ΔΔCT^ method (38).

The relative plasmid copy number was determined by comparing the quantification signals of the *bla_CTX-M-1_* (plasmid-encoded) and *garK* and *uidA* (chromosome-encoded) genes in SOS* to those in WT.

### Proteomics analysis

Proteomics analysis was performed as previously described with modifications (11, 39). The WT and SOS* were grown in LB without or with antibiotics (0.004 µg/mL CIP or 128 µg/mL CTX) to mid-exponential phase. Bacterial pellets were resuspended in 150 µL lysis buffer (7 M urea/thiourea mix [7:2 proportions], 20 mM dithiothreitol, and 2% sodium deoxycholate in 40 mM 4-(2-hydroxyethyl)-1-piperazineethanesulphonic acid buffer). Cells were disrupted through five cycles of sonication, each lasting for 10 s with 100% amplitude, with 25 s intervals of cooling in an ice/ethanol bath between cycles. Subsequently, the samples were shaken at 1,000 rpm for 1 h at room temperature, followed by centrifugation at 20,000 × *g* for 10 min at RT, after which the supernatants were collected.

Protein concentrations were determined using the Bradford protein assay (40), and 50 µg of protein from each sample (brought to the same volume across all samples with lysis buffer) was alkylated with 45 mM iodoacetamide (Sigma-Aldrich) for 40 min at room temperature in the dark. The pH of each sample was adjusted to 8.5 using 1 M triethylammonium bicarbonate, followed by protein digestion with 0.002 AU of lysC for 2 h at 37°C and 1 µg of trypsin for 4 h at 37°C.

Afterward, samples were labeled with one of the TMTpro 18-plex isobaric tags (Thermo Fisher Scientific) for 1.5 h, after which the reaction was quenched by hydroxylamine (Merck, Denmark). Labeled peptides were mixed 1:1 (reporter ion total signal), and the mix was reverse phase purified using an Oasis HLB cartridge (1 cc [10 mg]; Waters). Dried down purified peptides were dissolved in 20 mM ammonium formate, pH 9.3, loaded on an Acquity UPLC -Class CSH C18 column (Waters, USA), and high pH reverse phase separated into 12 fractions. A Dionex Ultimate 3000 HPLC system was used (Thermo Scientific, USA). Dried down peptide fractions were dissolved in 0.1% formic acid (FA; solvent A) and reverse phase separated by gradual increase of solvent B (95% acetonitrile [ACN], 0.1% FA, flow 250 nL/m) prior to detection by an Orbitrap mass spectrometer. The chromatographic column was pulled tip fused silica (75 um ID, 20 cm long) capillary, filled up with ReproSil-Pur 120 C18- AQ 1.9 µm (Dr. Maisch GmbH, Germany). Easy nLC 1000 (Thermo Fisher Scientific, Denmark) served as an HPLC instrument. Orbitrap Exploris 480 (Thermo Fisher Scientific, Germany) was used to detect and measure peptides operating under the following parameters. Total analysis time was 125 min with a 2 s cycle time (single full MS followed by DDA MS2 scans). Full MS: scan range 350–1,600 m/z at 120,000 resolution, positive mode, injection time set to “Auto.” MS2: precursor ions were selected for fragmentation at 1 × 10^4^ minimum intensity and charge state 2–5, subsequently put on the dynamic exclusion list for 45 s. Isolation window set to 0.7 m/z, HCD-fragmentation with normalized collision energy set to 34, Orbitrap resolution 45,000, and the first mass fixed to 110 m/z.

### Protein identification and quantification

Protein identification and quantification were performed using Proteome Discoverer, version 2.5 (Thermo Fisher Scientific). The search was performed against the database comprising the *E. coli* strain K-12 substr. MG1655 with pTF2 (GenBank: NC_000913.3, NC_032100.1, retrieved from Uniprot) using the Mascot search engine. Search parameters were enzyme trypsin/P, two missed cleavages, 5 ppm mass tolerance for MS, and 0.03 Da for MS2, variable modifications: TMTpro (K), TMTpro (N-terminus), carbamidomethyl (C), and oxidation (M). Peptides were quantified, allowing for isolation interference at a maximum of 30%, and proteins were normalized based on total peptide amount.

### SOS box prediction

To identify potential LexA-regulated genes encoded by plasmid pTF2 (GenBank: NC_032100.1), BLASTn searches were employed using **CTG**NNTNNNNNNN**CAG**, **TTG**DNTDNNHNNH**CAG**, and **CTG**DNTDNNHNNH**CAA** (1). To evaluate their binding affinity with LexA, the HI for each candidate sequence was calculated: heterology index = ∑ ln[(*n*_(consensus)_ + 0.5)/(*n*_(actual)_ + 0.5)], where *n*_(consensus)_ denotes the frequency of the consensus base at a given position among known LexA binding sites, and *n*_(actual)_ represents the frequency of the observed base at the same position in the candidate sequence (12). A lower HI value indicates a sequence more closely aligned with the consensus SOS box, implying a higher affinity for LexA binding. Conversely, an HI value greater than 15 suggests that the sequence is unlikely to bind LexA protein. The localization prediction of protein 140 was performed using SignalP 5.0, and the structure of protein 140 was predicted using AlphaFold2 implemented in ColabFold v1.5.5.

### Statistical analysis

Statistical analysis was done using GraphPad Prism. The comparisons between conjugation frequencies and gene expression levels were performed by one-way analysis of variance with Dunnett correction. Statistical significance was set to a *P*-value of ≤0.05.

## Data Availability

Proteomic data are deposited at DOI:10.17894/ucph.b4a308f9-6138-486e-9b46-f935011c3300. The mass spectrometry proteomics data have been deposited in the ProteomeXchange Consortium via the PRIDE [1] partner repository with the datasetdata set identifiers PXD063721 and https://dx.doi.org/10.6019/PXD063721.

## SUPPLEMENTAL MATERIAL

Supplemental figures spectrum.00090-25-s0001.docx Figures S1 to S5.

Supplemental tables spectrum.00090-25-s0002.docx Tables S1 and S2.

ASM does not own the copyrights to Supplemental Material that may be linked to, or accessed through, an article. The authors have granted ASM a non-exclusive, world-wide license to publish the Supplemental Material files. Please contact the corresponding author directly for reuse.
