# Supplementary material for: Mechanistic divergence between SOS response activation and antibiotic-induced plasmid conjugation in Escherichia coli
Source: Microbiol Spectr. 2025 May 28;13(7):e00090-25. doi: 10.1128/spectrum.00090-25 (PMC12211044; doi:10.1128/spectrum.00090-25)
Supplement: Supplemental figures — Figures S1 to S5. [file spectrum.00090-25-s0001.docx]

**Supplementary Figures**


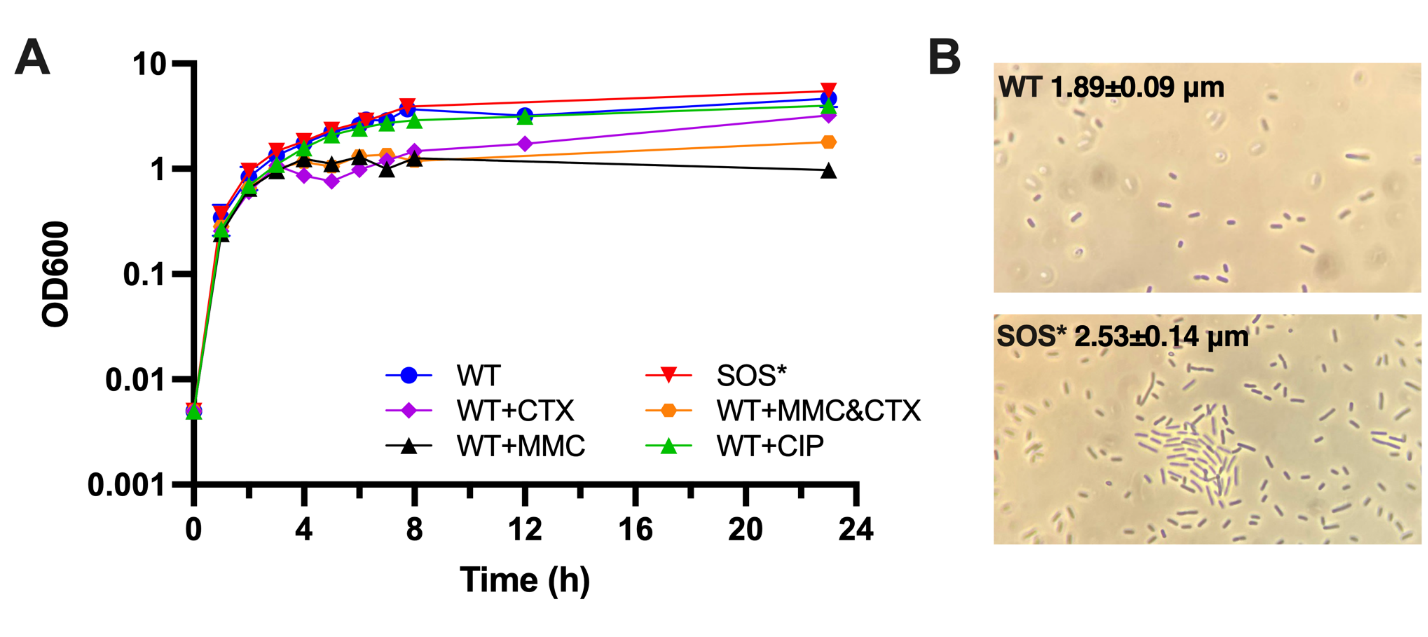


Figure S1. (A) Growth curve of WT and SOS* strain in LB medium. WT was treated with ½ MIC of antibiotic: CTX (128 μg/mL), MMC (2 μg/mL), combination of CTX (32 μg/mL) and MMC (1 μg/mL), and CIP (0.004 μg/mL) antibiotics were added at time 0 (B) Morphological observation of WT and SOS*. Bacteria were observed after 4 hours of growth in LB medium. The average cell length and standard errors are indicated.


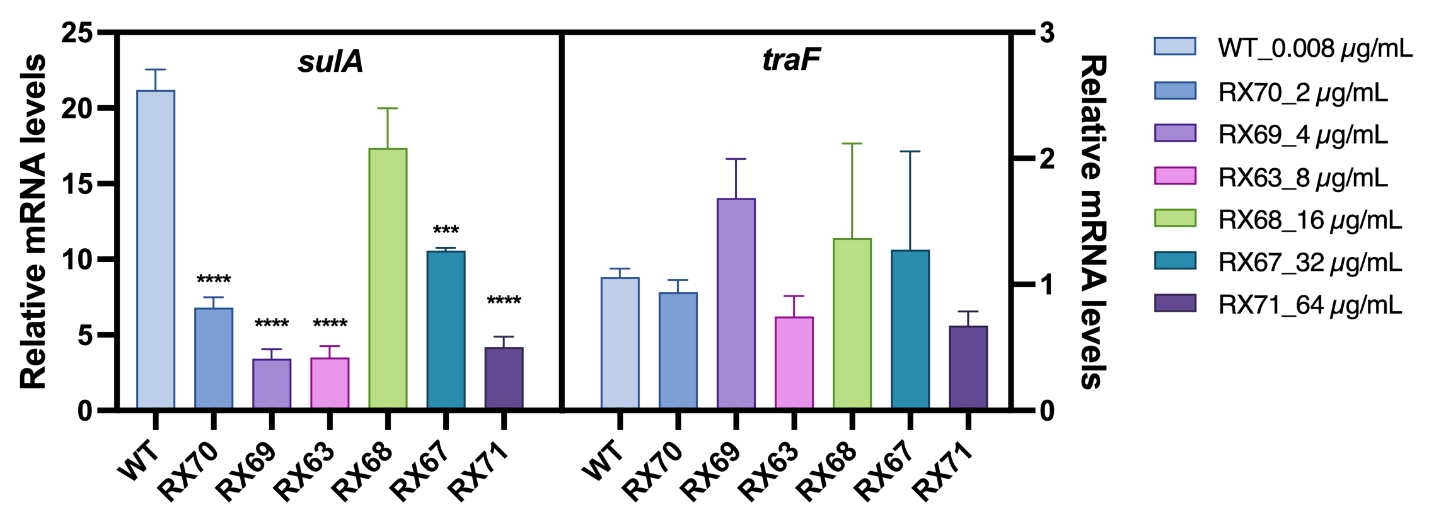


Figure S2. SOS response (*sulA*) and transfer-related (*traF*) gene expression levels in ciprofloxacin resistance strains exposed to ½-MIC CIP. Data are presented as fold change of gene expression in CIP treated strains relative to the corresponding strain without CIP treatment. The values are presented as average plus standard deviation. The legend shows the strain number and their respective CIP MIC levels. The fold changes were compared to that of WT to analyze the differences: ∗∗∗P ≤ 0.001, and ∗∗∗∗P ≤ 0.0001.


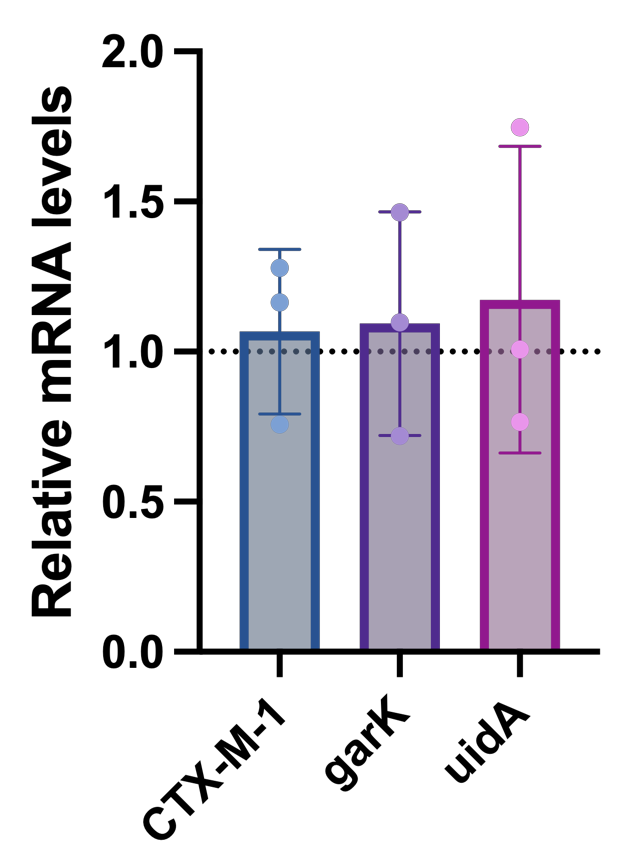


Figure S3. Investigation of relative plasmid copy number in the SOS* strain. The expression levels of the pTF2-encoded gene *bla_CTX-M-1_* and the MG1655 chromosome-encoded genes *gark* and *uidA* in SOS* were similar to those in WT. Data are presented as fold change relative to WT (dotted line) with the values presented as average plus standard deviation.


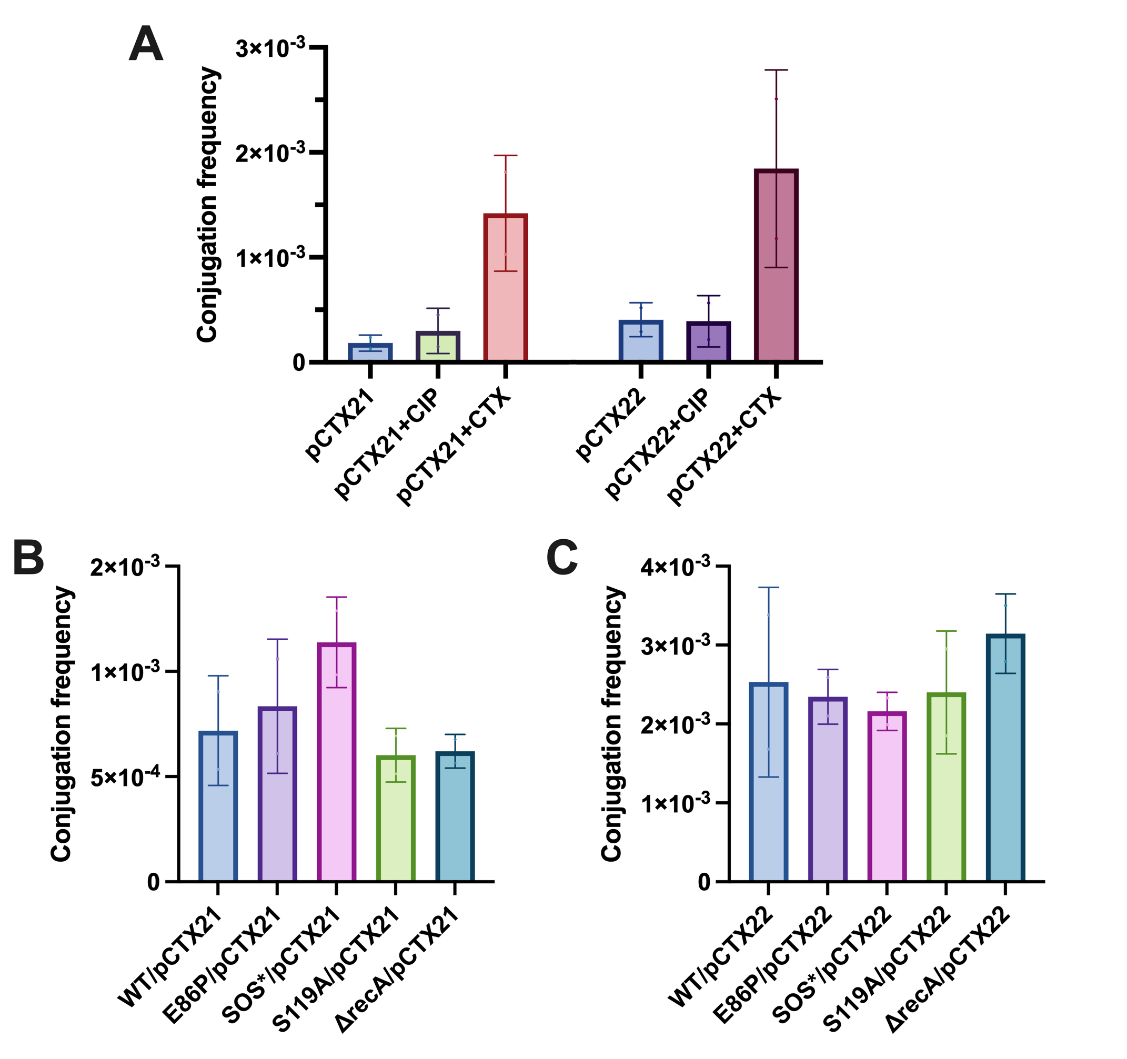


Figure S4. Conjugation frequency analysis of pCTX21 and pCTX22. (A) CTX treatment induces conjugation, while CIP does not. (B and C). Conjugation frequency of pCTX21 and pCTX22 remains unaffected by SOS mutations.


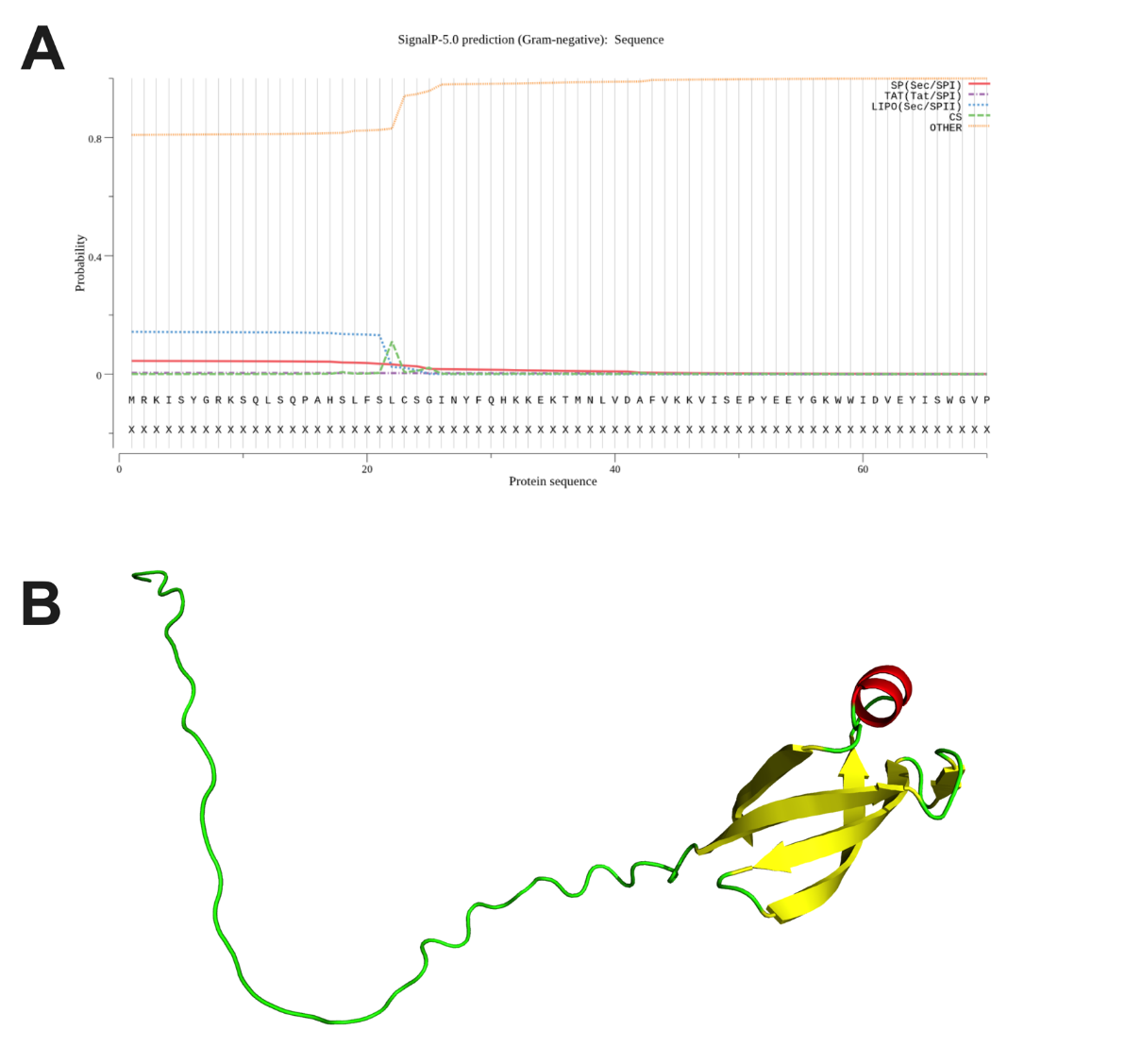


Figure S5. (A) Signal peptide prediction of 140 by using SignalP 5.0. (B) The structure of 140 was predicted using the AlphaFold2 algorithm implemented within ColabFold v1.5.5 and visualized by Pymol 3.0.3. The N-terminal consists of a 39-amino acid flexible and disordered region, while the C-terminal forms a mixed α/β domain.
