# Supplementary material for: Mechanistic divergence between SOS response activation and antibiotic-induced plasmid conjugation in Escherichia coli
Source: Microbiol Spectr. 2025 May 28;13(7):e00090-25. doi: 10.1128/spectrum.00090-25 (PMC12211044; doi:10.1128/spectrum.00090-25)
Supplement: Supplemental tables — Tables S1 and S2. [file spectrum.00090-25-s0002.docx]

**Table S1. Primers used in this study**

| **Application** | **Primer** | **Sequence (5'-3')** |
| --- | --- | --- |
| FRT variant construction | FRT-F | GGGGTTATTCGCAACGGCGACTGGCTGTAACATATCTCTGGTGTAGGCTGGAGCTGCTTC |
|  | FRT-R | GATGAAAAACAAACCGCGACGCCAGGCGGCATCGCGGTCTCATATGAATATCCTCCTTAG |
| SOE PCR for *lexA* E86P construction | lexA-F1 | TCGTCGCCGTGATTACCTGGTG |
|  | lexA E86P-R1 | GAAGTGGTGGACCGGCAGCCACACGACC |
|  | lexA E86P-F2 | CTGCCGGTCCACCACTTCTGGCGCAACAG |
|  | lexA-R2 | GCCATGCAGACAAGCCTGATGA |
| SOE PCR for *lexA* S119A and SOS* construction | lexA-F1 | TCGTCGCCGTGATTACCTGGTG |
|  | lexA S119A-R1 | TCTTTCATCGCCATCCCGCTGAC |
|  | lexA S119A-F2 | GTCAGCGGGATGGCGATGAAAGA |
|  | lexA-R2 | GCCATGCAGACAAGCCTGATGA |
| Δ*recA* construction | ΔrecA-F | TATTGACTATCCGGTATTACCCGGCATGACAGGAGTAAAAGTGTAGGCTGGAGCTGCTTC |
|  | ΔrecA-R | AAGGGCCGCAGATGCGACCCTTGTGTATCAAACAAGACGAATGGGAATTAGCCATGGTCC |
| Proof of kanamycin cassette insertion | id kan-R | CCTGCGCCATCAGATCCTTG |
| Proof of chloramphenicol cassette insertion | id Cm-R | CCACGACGATTTCCGGCAGT |
| Proof of *recA* knockout | idΔrecA-F | TCGTGCTGATTATGCCGTGT |
|  | idΔrecA-R | AGCTCCAGCGTGTCTTAACC |
| Δ*140* construction | Δ140-F | CAGGGAGTTACCGCTTCCTGCCCTTTAACAAAAAGAGATCGTGTAGGCTGGAGCTGCTTC |
|  | Δ140-R | GGTATCTCAATGCGAAGAAATAATCTCCGTAGAAACCCCCATGGGAATTAGCCATGGTCC |
| Δ*impB* construction | ΔimpB-F | ACCAGAAGAAACGCTGAAAATATGTTCGGAGATGACTGAAGTGTAGGCTGGAGCTGCTTC |
|  | ΔimpB-R | TGTCACTCACGTGATACACAGGACGCGGGAGTTACGCTGAATGGGAATTAGCCATGGTCC |
| Δ*impA* construction | ΔimpA-F | ATGAGGCGTAGAAACTGTTGATATCAGCCAGTGCAAACATGTGTAGGCTGGAGCTGCTTC |
|  | ΔimpA-R | AGAAATCTGGCAGGACGACAGCTGGCTGCCTGCAGCATGAATGGGAATTAGCCATGGTCC |
| Δ*impC* construction | ΔimpC-F | CGCCTGAAGGGTCAGCCGGACGGTGATAAACGGTACTCATGTGTAGGCTGGAGCTGCTTC |
|  | ΔimpC-R | ACTGTATATACATACAGCAAAAGAAAAGGGAGATGAGAACATGGGAATTAGCCATGGTCC |
| Δ*impCAB* construction | ΔimpB-F | ACCAGAAGAAACGCTGAAAATATGTTCGGAGATGACTGAAGTGTAGGCTGGAGCTGCTTC |
|  | ΔimpC-R | ACTGTATATACATACAGCAAAAGAAAAGGGAGATGAGAACATGGGAATTAGCCATGGTCC |
| RT-qPCR for detection of conjugation-related genes | sulA-F | ATCAGCCCATGATGACGCAA |
|  | sulA-R | CCCAGATGCCTGAACCCATT |
|  | recN-F | CACGGCGCGTAAAATGGAAA |
|  | recN-R | ACCTGAGTTGATTCGCCGAG |
|  | traF-F | GACGTCGGAATTTCATTTGC |
|  | traF-R | TCCACACGCTGATATTTTGG |
|  | traM-F | GGAGTCAGAATGATGCAATGG |
|  | traM-R | AGGGAGGAGATCTGTGAACG |
|  | gapA-F | ACTGACTGGTATGGCGTTCC |
|  | gapA-R | GTTGCAGCTTTTTCCAGACG |
| RT-qPCR for plasmid copy number comparison | garK-F | GATGCGGTCTTCAGCGTATT |
|  | garK-R | AATGCTTCGTCCAACGTACC |
|  | CTX-M-1-F | GACTATGGCACCACCAACG |
|  | CTX-M-1-R | GCTTTCTGCCTTAGGTTGAGG |
|  | uidA-F | CGCCGATGCAGATATTCGTA |
|  | uidA-R | CTGCCAGTTCAGTTCRTTGT |

**Table S2. Proteomic analysis- List of proteins encoded by pTF2.**

Ratio shown as number and color-coded


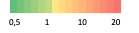


| **Locus_tag** | **Gene** | **Protein** | Abundance Ratio: SOS*/WT | Abundance Ratio: WT/WT+CIP | Abundance Ratio: WT/WT+CTX | Protein FDR Confidence: Combined |
| --- | --- | --- | --- | --- | --- | --- |
| HTE32_RS00020 | psiB | conjugation system SOS inhibitor PsiB | 1,298 | 1,011 | 1,116 | High |
| HTE32_RS00025 | psiA | plasmid SOS inhibition protein A | 1,026 | 0,916 | 1,037 | High |
| HTE32_RS00030 |  | hypothetical protein | 0,991 | 1,038 | 0,756 | High |
| HTE32_RS00050 |  | Rpn family recombination-promoting nuclease/putative transposase | 1,095 | 1,02 | 0,797 | High |
| HTE32_RS00055 |  | hypothetical protein | 0,912 | 0,963 | 0,677 | High |
| HTE32_RS00060 |  | helix-turn-helix transcriptional regulator | 1,196 | 1,309 | 1,156 | High |
| HTE32_RS00075 |  | molybdopterin-guanine dinucleotide biosynthesis protein MobC | 1,116 | 1,006 | 1,439 | High |
| HTE32_RS00080 | nikA | IncI1-type relaxosome accessory protein NikA | 1,084 | 1,104 | 1,012 | High |
| HTE32_RS00090 |  | Hypothetical protein | 1,078 | 0,969 | 1,068 | High |
| HTE32_RS00095 |  | peptidase domain-containing ABC transporter | 1,055 | 0,943 | 1,166 | High |
| HTE32_RS00100 |  | HlyD family efflux transporter periplasmic adaptor subunit | 1,086 | 0,856 | 0,989 | High |
| HTE32_RS00105 |  | CPBP family intramembrane metalloprotease | 1,002 | 1,07 | 0,67 | Medium |
| HTE32_RS00115 | TrbC | F-type conjugative transfer protein TrbC | 1,335 | 1,128 | 1,658 | High |
| HTE32_RS00120 | trbB | IncI1-type conjugal transfer protein TrbB | 1,638 | 0,979 | 3,375 | High |
| HTE32_RS00125 | trbA | IncI1-type conjugal transfer protein TrbA | 1,232 | 1,15 | 1,586 | High |
| HTE32_RS00135 |  | hypothetical protein | 1,244 | 1,033 | 1,303 | High |
| HTE32_RS00140 |  | hypothetical protein | 2,124 | 1,076 | 2,031 | High |
| HTE32_RS00145 |  | HEAT repeat domain-containing protein | 1,078 | 0,92 | 0,732 | High |
| HTE32_RS00150 | excA | plasmid IncI1-type surface exclusion protein ExcA | 1,145 | 0,98 | 2,274 | High |
| HTE32_RS00155 |  | DotA/TraY family protein | 1,602 | 1,065 | 2,554 | High |
| HTE32_RS00160 | traX | IncI1-type conjugal transfer protein TraX | 0,999 | 1,015 | 2,754 | High |
| HTE32_RS00165 | traW | IncI1-type conjugal transfer protein TraW | 1,51 | 1,044 | 2,354 | High |
| HTE32_RS00170 | traV | IncI1-type conjugal transfer protein TraV | 1,149 | 1,23 | 1,147 | High |
| HTE32_RS00175 | traU | IncI1-type conjugal transfer protein TraU | 1,451 | 1,049 | 2,483 | High |
| HTE32_RS00180 | traT | IncI1-type conjugal transfer protein TraT | 1,512 | 1,029 | 1,519 | High |
| HTE32_RS00190 | traR | IncI1-type conjugal transfer protein TraR | 2,875 | 1,352 | 3,399 | High |
| HTE32_RS00195 | traQ | conjugal transfer protein TraQ | 2,475 | 0,946 | 4,637 | High |
| HTE32_RS00200 | traP | IncI1-type conjugal transfer protein TraP | 2,063 | 1,105 | 3,686 | High |
| HTE32_RS00205 | traO | conjugal transfer protein TraO | 1,646 | 1,046 | 3,737 | High |
| HTE32_RS00210 | traN | IncI1-type conjugal transfer protein TraN | 1,614 | 1,011 | 4,784 | High |
| HTE32_RS00215 |  | DotI/IcmL family type IV secretion protein | 1,754 | 1,051 | 3,857 | High |
| HTE32_RS00220 |  | conjugal transfer protein | 1,173 | 1,023 | 1,137 | High |
| HTE32_RS00225 |  | DUF5710 domain-containing protein | 1,882 | 1,065 | 2,381 | High |
| HTE32_RS00230 |  | phospholipase D family protein | 1,76 | 0,972 | 1,095 | High |
| HTE32_RS00240 | traJ | plasmid transfer ATPase TraJ | 1,686 | 1,062 | 1,856 | High |
| HTE32_RS00245 | traI | IncI1-type conjugal transfer lipoprotein TraI | 1,962 | 1,041 | 3,26 | High |
| HTE32_RS00250 | traH | IncI1-type conjugal transfer lipoprotein TraH | 1,632 | 0,993 | 3,198 | High |
| HTE32_RS00255 |  | histidine phosphatase family protein | 1,71 | 1,126 | 3,558 | High |
| HTE32_RS00260 | TraF | conjugal transfer protein TraF | 2,161 | 1,002 | 2,856 | High |
| HTE32_RS00265 | TraE | conjugal transfer protein TraE | 1,629 | 1,116 | 1,946 | High |
| HTE32_RS00275 |  | shufflon system plasmid conjugative transfer pilus tip adhesin PilV | 1,155 | 1,048 | 4,064 | High |
| HTE32_RS00290 |  | type 4 pilus major pilin | 2,101 | 1,019 | 4,381 | High |
| HTE32_RS00295 |  | type II secretion system F family protein | 1,378 | 1,092 | 2,201 | High |
| HTE32_RS00300 | tadA | ATPase, T2SS/T4P/T4SS family | 1,288 | 0,942 | 2,902 | High |
| HTE32_RS00325 |  | IS66-like element ISEc23 family transposase | 1,19 | 1,11 | 2,266 | High |
| HTE32_RS00335 |  | PilN family type IVB pilus formation outer membrane protein | 1,518 | 1,001 | 3,686 | High |
| HTE32_RS00345 | pilL | type IV pilus biogenesis lipoprotein PilL | 1,646 | 1,026 | 3,314 | High |
| HTE32_RS00350 |  | hypothetical protein | 1,99 | 0,947 | 1,901 | High |
| HTE32_RS003600 |  | extended-spectrum class A beta-lactamase CTX-M-1 | 1,104 | 0,946 | 0,652 | High |
| HTE32_RS00365 |  | IS1380-like element ISEcp1 family transposase | 1,011 | 0,999 | 1,448 | High |
| HTE32_RS00375 | TraC | conjugal transfer protein TraC | 1,071 | 0,917 | 5,243 | Medium |
| HTE32_RS00405 |  | hypothetical protein | 1,094 | 0,995 | 2,438 | High |
| HTE32_RS00410 |  | ProQ/FINO family protein | 1,136 | 1,084 | 1,165 | High |
| HTE32_RS00415 |  | AAA family ATPase | 1,197 | 1,139 | 1,684 | High |
| HTE32_RS00420 |  | hypothetical protein | 1,055 | 1,058 | 1,132 | High |
| HTE32_RS00425 |  | hypothetical protein | 1,011 | 1,11 | 0,724 | High |
| HTE32_RS00435 |  | hypothetical protein | 1,059 | 1,034 | 1,31 | High |
| HTE32_RS00440 |  | site-specific integrase | 1,049 | 1,03 | 1,147 | High |
| HTE32_RS00445 |  | ParA family protein | 1,047 | 1,029 | 1,322 | High |
| HTE32_RS00450 |  | hypothetical protein | 1,034 | 0,981 | 0,992 | High |
| HTE32_RS00455 | ParM | plasmid segregation protein ParM | 1,034 | 1,002 | 1,445 | High |
| HTE32_RS00460 |  | plasmid partitioning/stability family protein | 1,058 | 1,054 | 0,889 | High |
| HTE32_RS00465 |  | Y-family DNA polymerase | 2,895 | 1,113 | 1,369 | High |
| HTE32_RS00470 | umuD | translesion error-prone D polymerase V autoproteolytic subunit | 21,333 | 1,588 | 1,49 | High |
| HTE32_RS00475 |  | DinI-like family protein | 8,652 | 1,715 | 0,88 | High |
| HTE32_RS00505 |  | hypothetical protein | 1,059 | 0,981 | 1,277 | High |
